# A Bayesian mixture model for Poisson network autoregression

**DOI:** 10.1007/s13278-025-01485-0

**Published:** 2025-07-17

**Authors:** Elly Hung, Anastasia Mantziou, Gesine Reinert

**Affiliations:** 1 Department of Statistics, University of Warwick, Coventry, CV4 7AL UK; 2 Department of Statistics, University of Oxford, Oxford, OX1 3LB UK

**Keywords:** Network regression, Poisson model, Bayesian mixture model, COVID-19

## Abstract

Multivariate count time series arise in a wide range of applications, including the number of COVID-19 cases recorded each week in different counties of the Republic of Ireland. In this example, it is natural to view the counties as nodes in a network, with edges between counties reflecting proximity. One could then model disease spread on a network through a regression model. Often Gaussian errors are assumed for such a model, but for count data this assumption may not be natural. With this motivating example in mind, we develop a model with the following features. We assume that the time series occur on the nodes of a known underlying network where the edges dictate the form of a structural vector autoregression model. In contrast to using a full vector autoregressive model, the network assumption is a means of imposing sparsity. Moreover we aim for a model that is able to accommodate heterogeneous node dynamics, and to cluster nodes that exhibit similar behaviour. To address these aims, we propose a new Bayesian Poisson network autoregression mixture model that we call a PNARM model, which combines ideas from Poisson network autoregression models, grouped network autoregression models, and non-uniform co-clustering priors.

## Introduction

Many real-world phenomena are recorded as a count time series, i.e., the observations are non-negative integers {0, 1, 2,...}, such as the number of disease infections in a district or discretisations of neural spike trains. The data often consists of multiple related time series: for example, commuters contribute to the spread of a disease across different districts (Seto et al. 2022), and reacting to stimuli requires co-ordination across multiple brain regions (Keeley et al. 2020). It is therefore helpful to model these time series jointly to characterise dependencies within and between these processes, and to identify similarities in their dynamics. Combining ideas from related literature, the goals of our proposed model are to induce sparsity through known network structures, which are related to associations between the time series, and to accommodate for heterogeneity between regions. An example which has motivated our approach is that of modelling COVID-19 data from Ireland. In Armbruster and Reinert (2024), a multivariate time series data set of weekly COVID-19 incidences for each of the 26 counties in the Republic of Ireland between 27 February 2020 and 23 January 2023 is analysed, spanning 152 weeks; we refer to this data set as the ‘Ireland COVID-19’ data. The data are separated into phases which reflect different regulations restricting physical movement and social interaction.

One of the most commonly used models for multivariate time series is the *vector autoregression* (VAR) model; however this model requires estimating a large number of parameters (Lütkepohl 2005). To deal with the high complexity of VAR models, a range of studies impose sparsity on the coefficients of the classic VAR model. A common shrinkage approach involves the specification of a Lasso-type penalty that restricts the number of parameters to be incorporated in each setting (Nicholson et al. 2020; Dallakyan et al. 2022). Alternative methods for imposing sparsity on the classic VAR model include the use of information criteria (Tsay 2013; Lütkepohl 2005), Bayesian shrinkage (Koop et al. 2019) and factor models (Forni et al. 2000).

For some applications, prior knowledge on the influence between different time series may be available, which motivates using network structure as a means of encoding dependence and inducing sparsity in these models. One of the earliest such models is the *network vector autoregression* model, proposed by Zhu et al. (2017), which assumes Gaussian noise. The proposed model in Zhu et al. (2017) for multivariate time series observed on the nodes of a fixed and known network assumes a network effect that uses the notion of immediate node neighbours as encoded by the observed network. This model is extended in Knight et al. (2020) to include higher-stages of node neighbours beyond immediate neighbours. In Martin et al. (2024) a network autoregressive model for networks with fixed weights and community structure is introduced. Instead of a time series observed on nodes, Mantziou et al. (2023) propose a network autoregressive model for time series observed on the edges; Mantziou et al. (2024) combine the notions of neighbouring nodes and neighbouring edges in a network autoregressive model tailored for time series observed on both the nodes and the edges of a fixed network.

A common assumption among all the aforementioned network autoregressive models is that of Gaussian noise. Especially when the node time series are time series of counts and when the values of the counts are low, an incorrect assumption of independent and identically distributed Gaussian errors can lead to estimators of the coefficients being inconsistent. Therefore, Armillotta and Fokianos (2024) propose a Poisson network autoregression model for count time series observed on the nodes of a fixed network with Poisson distributed responses.

Despite the wide applicability of these network autoregressive models, a key limitation is the assumption that the behaviour of the nodes is homogeneous. To address this limitation, Ren et al. (2024) propose a Bayesian mixture model for nodal time series assuming Gaussian noise. Similarly, Tao et al. (2023) propose a grouped network autoregressive model for count time series capturing the heterogeneity in the behaviour of the nodes using frequentist inference. Inspired by Ren et al. (2024), in this paper we introduce a Bayesian mixture model for Poisson network autoregression which allows inferences on the posterior distribution of the model parameters for count time series. We abbreviate this model as PNARM, for Poisson Network Auto-Regressive Mixture model. The PNARM model is a particular instance of a Bayesian mixture model with dependence. A survey of such models can be found in Wade and Inácio (2025).

To add more context for the PNARM model, in the literature, in contrast to our approach, time series of multivariate count data are sometimes analysed using graphical models. In graphical models, graphs represent conditional independence and relationships between variables. In network time series models, the network time series itself is the data. Our approach does not aim to construct a graphical model, but instead models variables on a graph. However, to provide a wider view of the field, here is a brief review of related approaches using graphical models. In Yang et al. (2013), Poisson graphical models are discussed; not only are all distributions on the nodes Poisson, conditional on the other nodes, but there is a unique joint distribution which is consistent with these node-conditional distributions. This unique distribution is a graphical model. Due to the stringent assumptions, this model only allows for negative dependence between the variables. Yang et al. (2013) introduce a truncation modification, which works under additional assumptions on the conditional dependencies. Dunson (2001) propose instead COunt Nonparametric Graphical Analysis (CONGA) which avoids restrictions on the dependence or truncation.

A general overview on Bayesian methods for multivariate count data can be found in Soyer and Zhang (2022), including also multivariate Poisson time series and dynamic latent factor models. For multivariate Poisson time series, the observations are assumed to follow a multivariate Poisson distribution, with particular emphasis on modelling the dependence between the Poisson variables. However statistical inference beyond the bivariate case is challenging. Dynamic latent factor models are a very flexible framework but would need careful fine tuning. Many more model choices are possible, see Soyer and Zhang (2022); here we propose a simple model, the PNARM model, which in our analysis provided a reasonable explanation of the data.

The main contributions of this paper are as follows;

1. a Bayesian version of the Poisson network autoregressive model in order to induce sparsity while being able to incorporate prior information,
2. reflecting heterogeneity in the network through a cluster structure in this model;
3. a new analysis of the Ireland COVID-19 data.

The remainder of this paper is organised as follows. Section 2 provides a more technical background to the relevant models from the introduction. Section 3 introduces the PNARM model and a method for performing inference. Simulations are provided in Sect. 4. Section 5 contains an application to the Ireland COVID-19 data. We mention some potential future research directions in Sect. 6. The code for the implementations can be found at https://github.com/ekhung/PNARM.

## Background

We begin by setting up some notation. We assume we have *N* time series, each with *T* time-steps, and use $Y_{i, t}$ to denote the random variable associated with the *i*-th univariate time series at time point *t*, for $i = 1,...,N$, and $t \in \mathbb {N}$. We refer to the *i*th time series as ${\textbf{Y}}_{i}$, the set of other time series as $\textbf{Y}_{-i}$ and denote by $\textbf{Y}_t$ the random vector at time *t*, with $\textbf{Y}_{1:T}$ referring to the vectors from time $t=1,...,T$. We are given a network on *N* nodes with adjacency matrix $\textbf{A}=(A_{i,j})_{i,j=1, \ldots , N}$. For a node *i* we denote its degree by $\textrm{deg}(i)$.

The following Poisson-distributed version of the network autoregression model was proposed in Armillotta and Fokianos (2024):

### Definition 1

*(Linear PNAR(1))* The linear Poisson network autoregression model of order 1, PNAR(1), is given by:

1 $$\begin{aligned} & Y_{i, t}| {\mathcal {F}}_{t-1} \sim \textrm{Poisson} (\lambda _{i, t}), \nonumber \\ &  \lambda _{i, t} = \beta _1 + \beta _2 \frac{1}{\textrm{deg}(i)} \sum _{j=1}^N A_{i, j} Y_{j, t-1} + \beta _3 Y_{i, t-1}, \end{aligned}$$

where $\mathcal {F}_{t-1}:= \sigma (\textbf{Y}_s: s \le t-1)$ is the $\sigma$-field generated by the collection of all events observable until time $t-1$. The coefficients $\beta _1, \beta _2, \beta _3$ are respectively referred to as the baseline, network effect and nodal effect coefficients, and are assumed to be positive to ensure positive $\lambda _{i, t}$ values.

In particular, the error terms of model (1) are not assumed to be identically distributed: conditional on the past, each random variable $Y_{i, t}, i=1, \ldots , N$ is assumed to be (marginally) Poisson distributed with possibly different parameters, and the joint distribution of $(Y_{i, t}, i=1, \ldots , N)$ depends on a copula function. However, a limitation is that the positivity constraint on the parameters only allows for modelling positive autocorrelation and cross-correlation. This constraint can be relaxed through using the canonical link for Poisson generalised linear models; however, under a log-linear link the predictors would have a multiplicative rather than additive effect on the expected value of the response variable.

By modelling the individual time series with the same coefficients $\beta _1, \beta _2, \beta _3$, the PNAR(1) model (1) assumes that the nodes are homogeneous. To allow for more flexibility, our work assumes that there are underlying clusters of nodes that are fairly homogeneous in their patterns, and consider a mixture model corresponding to distinct sets of baseline, network effect, and nodal effect coefficients to make inference about these clusters. A closely related work is the grouped network Poisson autoregression model, which extends the PNAR(1) model (1) by estimating cluster memberships for a fixed number of clusters through an expectation-maximisation algorithm (Tao et al. 2023). Unlike Tao et al. (2023), our proposed model is a Bayesian mixture model, in a similar vein to the *graphical assistant grouped network autoregression model* (GAGNAR) (Ren et al. 2024). The hierarchical model for GAGNAR is

2 $$\begin{aligned} \textbf{Z} &\sim \text {gaCRP}(\alpha , h), \\\sigma ^2_{k} &\sim \text {Inverse-gamma}(a_0, b_0), \\ \varvec{\theta }_k&\sim \text {MVN}(\mu _0, \sigma ^2_{k} \Sigma _0), \\Y_{i, t} \mid {\mathcal {F}_{t-1}}, z_i, \varvec{\theta }_{z_i}, \sigma ^2_{z_i} &\sim \text {N} \left( \theta _{1, z_i} + \theta _{2, z_i} \frac{1}{\textrm{deg}(i)} \sum _{j=1}^N A_{i, j} Y_{j, t-1} + \theta _{3, z_i}, Y_{i, t-1}, \sigma _{z_i}^2 \right) , \end{aligned}$$

where $\textbf{Z} = (Z_1, \dots , Z_n)$ is a vector of the cluster memberships of the nodes, and the error structure satisfies the conditional independence relationship $Y_{i, t} \perp Y_{j, t} \mid \textbf{Z}, {( \varvec{\theta }_k)_k, (\varvec{\sigma }^2_k)_k} , \mathcal {F}_{t-1}$ for any $i \ne j$. The prior over partitions gaCRP$(\alpha , h)$, which Ren et al. (2024) call a ‘graphical assistant Chinese Restaurant Process’, is similar to the *distant-dependent partition prior* by Dahl (2008) given in (6) below, but without normalising the weights $h_{ij}$. To incorporate network information, Ren et al. (2024) select $h_{ij}$ to depend on $d_{ij}$, the shortest path length between node *i* and *j*, with

3 $$\begin{aligned} h_{ij}= {\left\{ \begin{array}{ll} 1, & d_{ij} \le 1 \\ \exp (- h \times d_{ij}), & d_{ij} > 1, \end{array}\right. } \end{aligned}$$

where *h>0* is a parameter. Many more choices of priors and variations of Dirichlet process priors for multinomial probabilities are available; see for example Moraffah (2024) for a survey. Some details on Bayesian analysis can be found in Appendix A.4.

## Method

Our Poisson network autoregression mixture model (which we abbreviate to PNARM model) assumes that nodes have a latent class label which affects its autoregressive properties. The class assignment itself is random as well. We view the classes as clusters of fairly homogeneous nodes. Mathematically, let $Z_i$ denote the latent class of node *i* and let $\textbf{Z} = (Z_1, Z_2, ..., Z_N)$, $K(\textbf{Z}) = \max _i Z_i$ so that each $Z_i \in \{1, .., K(\textbf{Z})\}$. Each cluster *k* is given an associated parameter triplet ${\varvec{\theta }}_k = (\theta _{1,k}, \theta _{2,k}, \theta _{3,k})$; we abbreviate ${\varvec{\theta }} = ({\varvec{\theta }}_k, 1 \le k \le K(\textbf{Z})).$

The PNARM model is given in hierarchical form as

4 $$\begin{aligned}\textbf{Z} = (Z_1, \ldots , Z_N) &\sim \pi _\textbf{Z} \\{\varvec{\theta }}_k & \overset{\text {i.i.d.}}{\sim} \pi _{\varvec{\theta }} \text { for } k = 1, \dots , K(\textbf{Z})\\\quad p(\textbf{Y}_i \mid \mathbf {Z, {\varvec{\theta }}, {Y}}_{-i}) &\propto \prod _{t=2}^T \text {Poisson}\left( Y_{i, t}; \lambda _{i, t, \varvec{\theta }_{Z_i}} \right) \\ \lambda_{i, t, \varvec{\theta }_{Z_i}} &:= \theta _{1, Z_i} v_i + \theta _{2, Z_i} X_{i, t-1} + \theta _{3, Z_i} Y_{i, t-1} \end{aligned}$$

where $v_i { > 0 }$ is a known constant associated with each node that is fixed in time, and $X_{i, t}$ may depend on the past observations at other nodes, e.g. as in (5) below. The error structure also satisfies the conditional independence relationship $Y_{i, t} \perp Y_{j, t} \mid \varvec{Z, \theta }, \mathcal {F}_{t-1}$ for any $i \ne j$. The prior $\pi _\textbf{Z}$ is on the latent class allocations, which determines the partition of the nodes, and $\pi _{\varvec{\theta }}$ is a prior for the autoregression coefficients. The rate $\lambda _{i, t, \varvec{\theta }}$ is the expected value of the observation for node *i* at time *t* conditional on $\varvec{\theta }$ and on the process up to time $t-1$.

The PNARM model extends the GAGNAR model of Ren et al. (2024) from Gaussian to Poisson distributed observations and generalises the prior for the partition on the nodes. The linear Poisson network autoregression PNAR(1) model by Armillotta and Fokianos (2024) in (1) is a frequentist special case of (4), with $v_i$ and $X_{i, t}$ as generalisations of the intercept and network effect predictors in (1). Our model may be extended to a higher lag-order in an analogous way to VAR models, but for the remainder of the paper, we focus on lag-1 models.

Instead of using the same predictors as in (1), the PNARM model in (4) allows $v_i$ and $X_{i, t-1}$ to depend on known network information, which may help with modelling heterogeneous node dynamics. For example, to account for the difference in population sizes across different counties, one may apply a population adjustment, taking the predictors to be

5 $$\begin{aligned} v_i = {p_i}/{c} \quad \text {and} \quad X_{i, t-1} = \frac{p_i}{\text {deg}(i)} \sum _{j=1}^N A_{i, j}Y_{j, t-1} / {p_j}, \end{aligned}$$

where $p_i$ is the population size of county *i* and $c \in \mathbb {R}_{>0}$ is some fixed constant. When writing the autoregression in vector form, population adjustment is equivalent to replacing the adjacency matrix $\textbf{A}$ with $\textbf{PAP}^{-1}$, where $\textbf{P} = \text {diag}(p_1, p_2, ..., p_N)$. This can also be interpreted as converting the unweighted, undirected network with adjacency matrix $\textbf{A}$ into a weighted, directed network: every $i \sim j$ edge is replaced with a directed $i \rightarrow j$ edge weighted by ${p_j}/{p_i}$ and a directed $j \rightarrow i$ edge weighted by ${p_i}/{p_j}$.

In the remainder of the paper, we focus on two possible choices of partition prior $\pi _\textbf{Z}$: the Dirichlet-multinomial finite mixture model (FMM) with a non-informative prior for mixture proportions, and a distant-dependent partition prior (DDP) by Dahl (2008) which generalises the uniform co-clustering assumption in the Dirichlet process, by implicitly defining a prior through the co-clustering probabilities, as follows. Let $S({\textbf {Z}})$ denote a partition induced by the cluster labels ${\textbf {Z}}$, with $S_k$ being a particular element in the partition, and writing $S({\textbf {Z}}_{-i})$ to be the partition $S({\textbf {Z}})$ but with node *i* removed, the co-clustering probabilities are given by

6 $$\begin{aligned} p(i \in S_k \mid S({\textbf {Z}}_{-i})) \propto {\left\{ \begin{array}{ll} \sum _{j: j \ne i, \,j \in S(\mathbf{Z}_{-i})_k} h_{ij}, & k = 1,..., K(S({\textbf {Z}}_{-i})) \\ \alpha , & k=K(S({\textbf {Z}}_{-i}))+1, \end{array}\right. } \end{aligned}$$

where the weights $h_{ij}$ are scaled to satisfy $\sum _{j: j \ne i} h_{ij} = N-1$. The use of the partition notation is to emphasise that the model (6) is invariant to a permutation of the label indices. From (6), we have that the probability of forming a new cluster is $\frac{\alpha }{\alpha + N- 1}$, the same as in a Dirichlet process mixture model – setting $h_{ij} = 1$ for all *i*, *j* recovers the Dirichlet process prior. We set the co-clustering weights between *i* and *j* to be $h_{ij} \propto \exp (-h  d_{ij})$, where $d_{ij}$ is the shortest path between nodes *i* and *j* and $h$ is a parameter. This choice of $h_{ij}$ means that large values of *h* decrease the prior probability of a node being clustered with its neighbours, exponentially down-weighted for nodes that are further away. As $\alpha$ increases, the expected number of clusters increases.

As for the prior distribution $\pi _{\varvec{\theta }}$ on the model parameters $\theta _{1,k},\theta _{2,k},\theta _{3,k}$, $\text { for } k = 1, \ldots , K(\textbf{Z})$, a common choice of prior distribution for the rate of a Poisson distribution is a Gamma distribution due to conjugacy. In our setting, specifying a Gamma prior for each of the $\theta _{\cdot ,\cdot }$ parameters does not result in the rate of the Poisson being Gamma distributed as $\theta _{\cdot ,\cdot }$ can have different Gamma rates. Despite not being able to exploit conjugacy in our setting, we still consider Gamma priors for $\theta _{\cdot ,\cdot }$ as a Gamma distribution is a natural choice considering the support of the parameters (we assume that $\theta _{\cdot ,\cdot }> 0$, so that $\lambda _{\cdot ,\cdot ,\cdot }> 0$).

In the case that the clusters and autoregressive coefficients are known and fixed, we have the following sufficient condition for stationarity; the proof is deferred to Appendix A.1.

### Proposition 2

Given fixed node latent classes $\textbf{Z}$ and cluster coefficients $\varvec{\theta }$, a sufficient condition for stationarity of the PNARM(1) model (4) when the network effect predictor is the average of lagged neighbour values, i.e. $X_{i, t-1} = \frac{1}{\textrm{deg}(i)} \sum _{j=1}^N A_{i, j} Y_{j, t-1}$, is that

7 $$\begin{aligned} \max _{k=1,..., K } \theta _{2, k} + \theta _{3, k} < 1. \end{aligned}$$

In the case where the network effect predictor is adjusted for population, i.e. $X_{i, t-1} = \frac{p_i}{\textrm{deg}(i)} \sum _{j=1}^N A_{i, j} \frac{1}{p_j} Y_{j, t-1}$, a sufficient condition for stationarity is

8 $$\begin{aligned} \max _{k=1,..., K} \theta _{2, k} + \max _{k=1,..., K} \theta _{3, k} < 1. \end{aligned}$$

To target samples from the posterior of (4), since the posterior distribution of $\varvec{\theta }$ is not available in closed form we use a Markov Chain Monte Carlo (MCMC) algorithm that alternates between sampling cluster labels for the nodes via a Gibbs sampler and sampling coefficients for the clusters using a random-walk Metropolis–Hastings step. This is provided in Algorithm 1.

**Algorithm 1:**
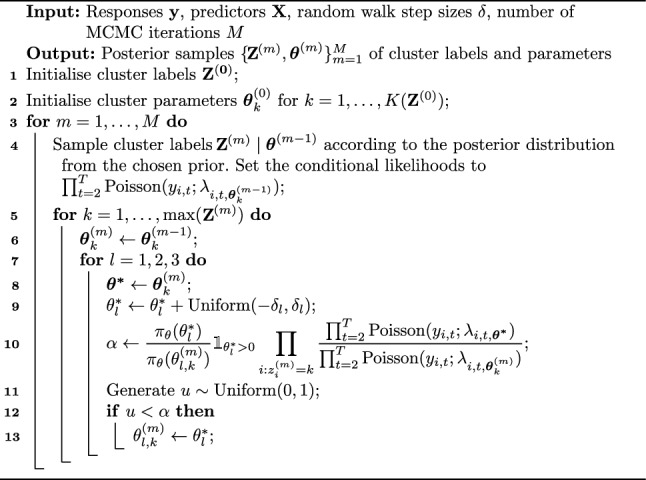
MCMC algorithm targeting the posterior of the coefficients of model (4)

One challenge of inference for mixture models is the ‘label switching’ identifiability problem: the likelihood of a mixture model is invariant under permutations of the cluster labels which may cause difficulties during inference (Jasra et al. 2005). Therefore, in what follows we focus on using the MCMC outputs in a label-invariant way.

Let *m* index the MCMC iteration, with $(\textbf{Z}^{(m)}, {\varvec{\theta }}^{(m)})$ being the *m*-th sample, *M* the total number of MCMC samples, and $S^{(m)}$ the partition induced by the cluster labels $\textbf{Z}^{(m)}$. Let $\hat{c}_{ij} := \frac{1}{M} \sum _{m=1}^M \mathbbm {1}_{z_i^{(m)} = z_j^{(m)}}$ denote the proportion of times that node *i* and *j* are allocated to the same cluster among the MCMC samples. As in Dahl (2008) we select a partition among the samples that minimises the loss function

9 $$\begin{aligned} S^{LS}:= \min _{S^{(1)}, S^{(2)},..., S^{(M)}} \sum _{i \ne j} (\mathbbm {1}_{z_i^{(m)} = z_j^{(m)}} - \hat{c}_{ij})^2. \end{aligned}$$

While the exact posterior predictive distribution is intractable, we may approximate it by considering node coefficients individually. For  Sample *m*, the coefficients associated with node *i* are $\theta _{l, z_i^{(m)}}^{(m)},$  for $l = 1, 2, 3.$ 

The behaviour of the node can be summarised into a point estimate via its $\textit{mean node coefficient}$,

10 $$\begin{aligned} \frac{1}{M} \sum _{m=1}^M {\varvec{\theta }}_{z_i^{(m)}}^{(m)}, \end{aligned}$$

which averages over all of the sampled partitions.

Letting $\Omega _S$ denote the set of all possible partitions of the set of nodes, the posterior predictive distribution is given by:

11 $$\begin{aligned} & p(\textbf{y}_{T+1} | \textbf{y}_{1:T}) \propto \sum _{S \in \Omega _S}\nonumber \\ & \quad \left( \pi _S(S) \prod _{k=1}^{K(S)} \int _{\mathbb {R}^3_{>0}} \pi _{\varvec{\theta }}(\varvec{\theta }_k) \prod _{i \in S_k} \text {Poisson}(y_{i, T+1}; \lambda _{i, T, \varvec{\theta }_k}) \, \textrm{d} \varvec{\theta }_k \right) \end{aligned}$$

where $\lambda _{i, t, \varvec{\theta }_k} := \theta _{1, k} v_i + \theta _{2, k} x_{i, t-1} + \theta _{3, k} y_{i, t-1}$. If the MCMC algorithm is ergodic and has stationary distribution equal to the posterior distribution $\pi (\varvec{S}, \varvec{\theta } | \textbf{y})$, then for large enough *M* a law of large number approximation of (11) can be obtained as a finite mixture of Poisson distributions:

12 $$\begin{aligned} & \hat{p}(\textbf{y}_{T+1} | \textbf{y}_{1:T}) = \frac{1}{M} \prod _{i=1}^N \sum _{m=1}^M\nonumber \\ & \quad \text {Poisson}\left( y_{i, T+1}; \,\theta _{1, z_i^{(m)}}^{(m)} w_i + \theta _{2, z_i^{(m)}}^{(m)} x_{i, T} + \theta _{3, z_i^{(m)}}^{(m)} y_{i, T} \right) . \end{aligned}$$

## Simulations

In this section, we show some simulation studies to see whether the MCMC algorithm recovers reasonable model fits, for a range of underlying networks and coefficients.

**Fig. 1:**
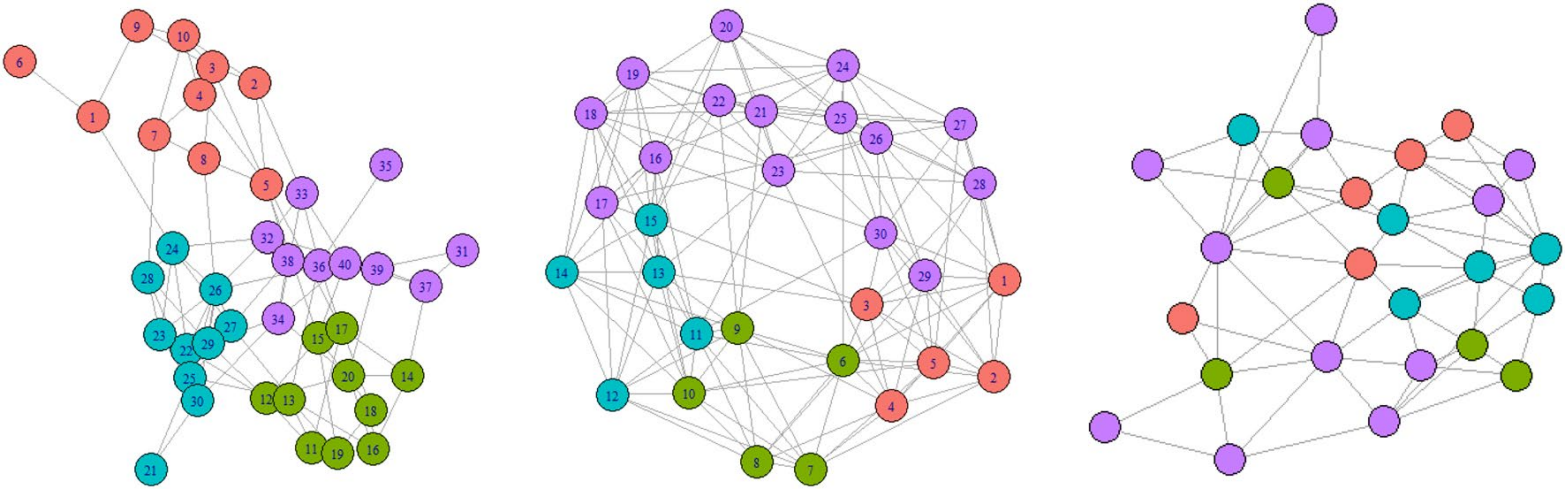
The three underlying networks considered in simulations: (left) generated from a stochastic block model, (middle) generated from a Watts-Strogatz model, and (right) the economic hubs network with 4 clusters

The first artificial network considered is a 40 node network generated from a 4-cluster stochastic block model with within-cluster edge probabilities 0.4 and between-cluster edge probabilities 0.05. The second artificial network is a 30 node network generated from a 1-dimensional Watts–Strogatz small-world model from Watts and Strogatz (1998) with size five neighbourhoods and a rewiring probability of 0.05, with cluster membership set as $\{1,..., 5\}, \{6,..., 10\}, \{11,..., 15\}$, and $\{16, ..., 30\}$. Finally, we also consider the ‘economic hubs network’ (described in Sect. 5.2) used to model the COVID-19 data from Armbruster and Reinert (2024), with population adjustment according to the associated population data. The clusters for the ‘economic hubs network’ simulations were set to the least-squares clustering (9) obtained from a trial run of the MCMC Algorithm 1 with $k=4$ components specified. These networks are shown in Fig. 1, where the node colours correspond to the cluster memberships of the nodes.

The coefficients used to simulate these time series can be found in Table 1; in particular, the coefficients in Example 3 are chosen to be similar to those obtained from the trial MCMC run on the COVID-19 data. For the coefficient configurations that meet the sufficient conditions for stationarity in Proposition 2, the first 100 samples were discarded as burn-in so that the simulated series is likely to be drawn from a distribution close to the limiting stationary distribution. For Example 3, the simulated time series were started from 0 to mimic the development of the number of COVID-19 cases.

**Table 1:** Coefficient values for the different simulation examples

	Example 1			Example 2			Example 3		
$\theta _{\text {type, cluster}}$	$\theta _{1, \bullet }$	$\theta _{2, \bullet }$	$\theta _{3, \bullet }$	$\theta _{1, \bullet }$	$\theta _{2, \bullet }$	$\theta _{3, \bullet }$	$\theta _{1, \bullet }$	$\theta _{2, \bullet }$	$\theta _{3, \bullet }$
$\theta _{\bullet , 1}$ (red)	0.5	0.1	0.2	1	0.3	0.1	1.8	1.5	0.1
$\theta _{\bullet , 2}$ (green)	0.8	0.2	0.3	1.5	0.1	0.8	1.3	0.7	0.01
$\theta _{\bullet , 3}$ (blue)	1.3	0.3	0.4	1	0.4	0.2	2.9	0.01	0.9
$\theta _{\bullet , 4}$ (purple)	1.7	0.4	0.5	1.5	0.2	0.5	0.9	0.1	0.9

For each example set of coefficients in Table 1 and network in Fig. 1, three different seeded time series were simulated on the nodes of the network. For each time series, the MCMC algorithm was run for 11 000 iterations, discarding the first 1000 iterations as burn-in. Throughout, the hyperparameters of the distant-dependent partition prior (DDP) are set to $\alpha =h=1$, the hyperparameters are set to Dirichlet((1, 1, 1, 1)) in the Dirichlet-multinomial finite mixture model (FMM), and the priors for the coefficients are set to $\Gamma (1, 1)$.

To assess how well the coefficients are recovered, we compute the mean square errors between the mean node coefficient and the true coefficient, which are listed in Table 2. We also compute the proportion of times that the true node coefficient lies within the 90% highest posterior density region estimated from the MCMC samples, as measured using the R package coda by Plummer et al. (2006); these can be found in Table 3. The metrics reported are from averaging over all nodes and all three simulated time series.

To assess how well the underlying partitions are recovered, we calculate the adjusted Rand Index (ARI) comparing the true clusters against the least-square clustering from the MCMC samples; these are in Table 4. The ARI is based on the number of pairs of objects that are clustered together in both partitions and is scaled to [−1, 1] so that an ARI of 0 is expected when the clustering is random and an ARI of 1 corresponds to perfect cluster recovery (Hubert and Arabie 1985).

**Table 2:** The MSEs of the mean node coefficients (10) compared to true coefficient values; lower values are better

	Example 1		Example 2		Example 3	
	DDP	FMM	DDP	FMM	DDP	FMM
SBM	0.10	0.13	0.06	0.07	0.61	0.84
Watts-Strogatz	0.06	0.12	0.07	0.08	0.18	0.18
Economic hubs	0.27	0.27	0.05	0.05	0.13	0.41

**Table 3:** Proportion of times the true node coefficient falls within the 90% HPD interval estimated from the MCMC samples; values closer to 0.9 are better

	Example 1		Example 2		Example 3	
	DDP	FMM	DDP	FMM	DDP	FMM
SBM	0.79	0.67	0.83	0.84	0.53	0.15
Watts-Strogatz	0.97	0.85	0.89	0.88	0.86	0.86
Economic hubs	0.64	0.63	0.75	0.83	0.88	0.56

**Table 4:** Average ARI comparing least-squares partitions to true clusters; higher values (maximum of 1) are better

	Example 1		Example 2		Example 3	
	DDP	FMM	DDP	FMM	DDP	FMM
SBM	0.38	0.27	0.45	0.43	0.85	0.31
Watts-Strogatz	0.81	0.76	0.52	0.43	0.67	0.72
Economic hubs	0.69	0.63	0.27	0.29	0.75	0.40

**Fig. 2:**
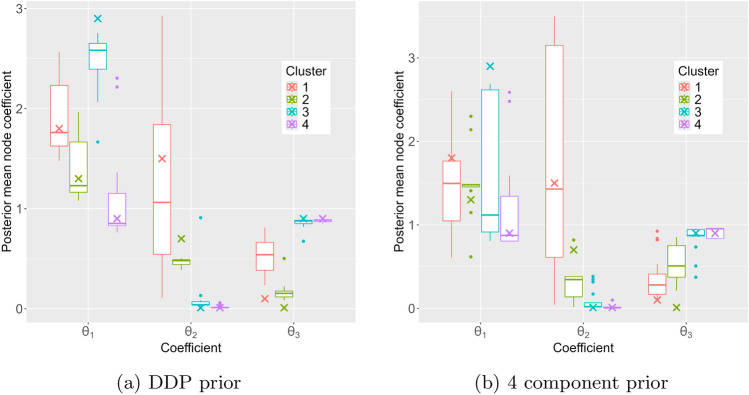
Boxplot of mean node coefficients, with true values in crosses, simulating with least-squares clusters and coefficients estimated from the data

While the distant-dependent prior favours assigning nodes that are close in network distance to the same cluster, which is true in the underlying networks, the 4-component mixture model matches the known true number of components. In the simulations, the sampler for the DDP prior generally recovered the node coefficients and node clusters more accurately and constructed more accurate 90% HDP intervals compared to the sampler for the FMM prior. The results also suggest that the ‘difficulty’ of recovering the data-generating process depend on both the network topology and the coefficients, as results vary even along the same rows and columns of Tables 2 to 4, and there is not necessarily a correspondence between accurate partition recovery and accurate coefficient recovery. Figure 2 shows boxplots of the mean node coefficients from (10) obtained in the simulations that are most relevant to the real data application in the next section.

## Application to COVID-19 case modelling

### Data description

From the end of February 2020 onwards, the Irish Health Protection Surveillance Centre published weekly updates to various COVID-19 related statistics from the Republic of Ireland. We use a data set which recorded the daily cumulative case count in each of the 26 counties in the Republic of Ireland (attributed by the patient’s location of residence) that can be obtained at Health Protection Surveillance Centre (2023). The daily number of new cases per county obtained from the data set were aggregated into the number of new cases reported weekly, as day-to-day variations in the case data are often subject to cyclical trends in testing within a week or short-term fluctuations such as lab delays and clustered reporting of data for several days (Sartor et al. 2020). Our data set starts on March 1, 2020, with the first recorded case on March 2, 2020, and ends on January 23, 2023, giving 26 time series of length 152 weeks.

### Previous work

**Fig. 3:**
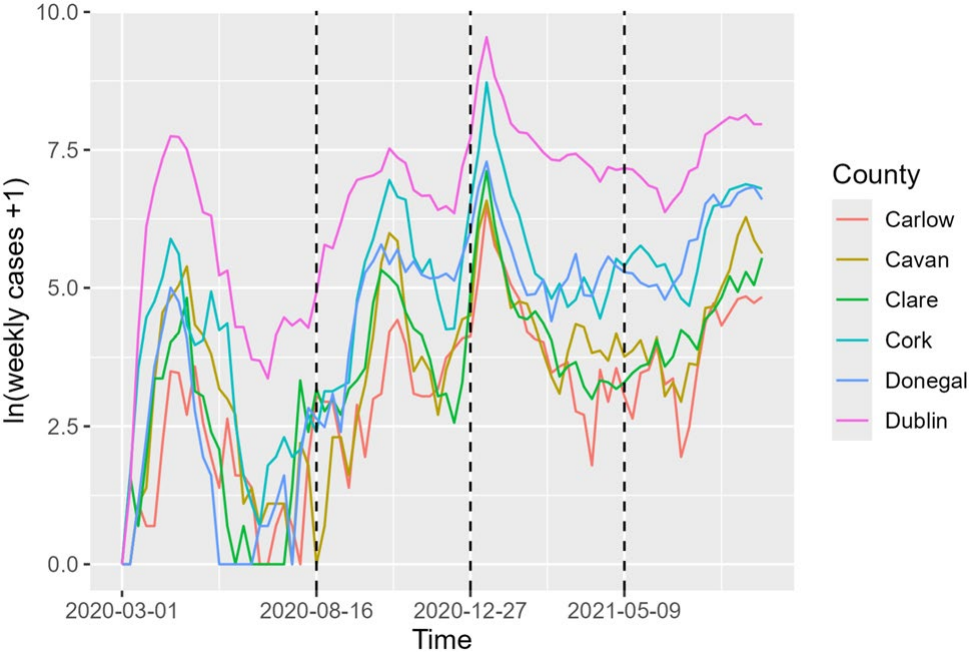
Log plot of (weekly cases + 1) for six counties, with segments indicated by dashed lines

**Fig. 4:**
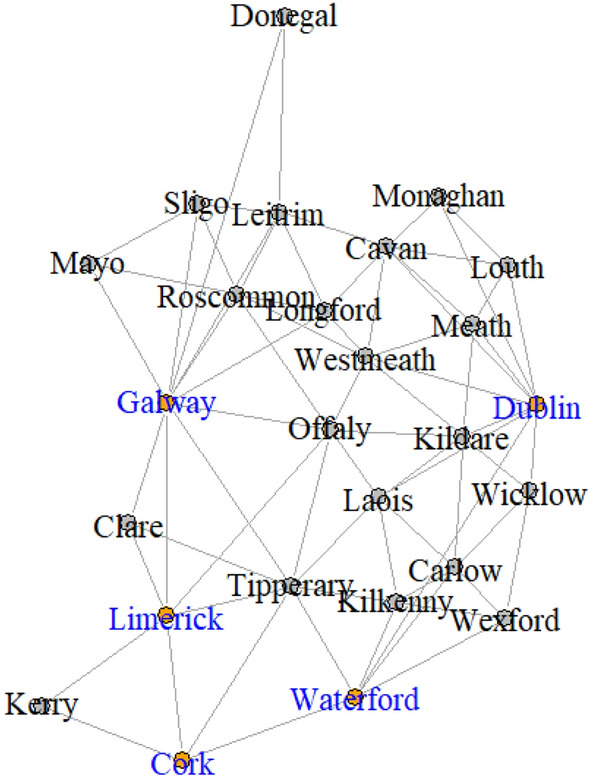
The economic hubs network, with hubs in blue, adapted from Armbruster and Reinert (2024)

To model the weekly number of cases, Armbruster and Reinert (2024) investigated network choice for fitting the generalised network autoregression model (Knight et al. 2020) to the Ireland COVID-19 data, splitting the full data into five segments based on the COVID-19 regulations governing inter-county travel and social interaction restrictions at the time. We focus on the first segment from the start of the pandemic, starting from 01/03/2020 and lasting for 25 weeks, as shown in Fig. 3, which is classified as being in the restricted phase. In Armbruster and Reinert (2024), the most promising results were found for the ‘economic hubs network’, which is shown in Fig. 4. They define the economic hubs network as follows: first connect each county to the counties it shares borders with; second, additional edges are added if needed to connect each county to its nearest economic hub: Dublin, Cork, Limerick, Galway, or Waterford, as a proxy for commuter flow, since commutes have been found to be significant for spatial dynamics of COVID-19 cases, such as in the study by Mitze and Kosfeld (2022). We include a comparison to the railway and complete networks of Armbruster and Reinert (2024) in Sect. A.3 of the Appendix.

### Modelling considerations

In principle it would be good practice to elicit a prior through expert opinions. For multivariate priors however this is not an easy task; Moala and O’Hagan (2010) give some guidance for Gaussian process priors, while Mikkola et al. (2024) surveys the problem. Hence we resort to priors which have been used in the literature.

In Laroze et al. (2021), fitting linear autoregressive models to COVID-19 data results in estimated coefficients corresponding to network effects and nodal effects between 0.18 and 1.6. While regional differences in policies and travelling patterns can lead to different contagion dynamics, a Gamma(1, 1) prior across all coefficients might be a promising starting point. Hence, in the comparisons, we use independent Gamma(1, 1) priors for each of the coefficients in the PNARM model. For some applications, it may be helpful to select prior distributions that have support constrained to coefficients that would lead to stationary processes; while this is not true of a Gamma prior distribution, stationarity may not be expected for our application to case incidence data in an epidemic stage. Lacking prior information, for the partition prior of the FMM we consider a Dirichlet(1,...,1) of order $K(\varvec{Z})$, which is a uniform prior and is a widely used non-informative prior for multinomial probabilities. In Sect. A.2 of the Appendix, results from some preliminary MCMC runs other parameter settings for the priors are also given; they suggest some robustness to prior choice.

### ‘Chain-stacking’

Insufficient exploration of the parameter space when using MCMC methods for mixture models has been well-documented in the literature (e.g. Marin et al. (2005)). To try to mitigate against invalid inference from non-mixing chains, we explored a similar chain stacking approach to Yao et al. (2022). While the procedure does not return the true posterior of the model, it may have more robustness against model misspecification when forecasting future values. Directly following the leave-one-out heuristics of Yao et al. (2022) is challenging for our application due to the sequential nature of time series data. Instead we leave some of the end time points out of the training data to use as a validation set; this procedure can be seen as a special case of the leave-future-out cross validation (LFO-CV) method of Bürkner et al. (2020). The proposed approximation is as follows: we run *C* parallel chains for fitting models to the data from time points 1 to $T_{train}$, and obtain estimates of the posterior distributions for ($\varvec{Z, \theta }$). Letting $\Delta ^{{C}-1}$ denote the probability simplex in $\mathbb {R}^C$, we consider weights defined as

13 $$\begin{aligned} & \mathbf {w^*} = \arg \max _{\textbf{w} \in \Delta ^{{C}-1}} \Bigg( \sum _{t=T_{train}+1}^T \nonumber \\ & \quad \log \sum _{c=1}^C w_c \hat{p}_c (\textbf{y}_t | \textbf{y}_{1:(t-1)}) + \log \text {Dirichlet}(\textbf{w}; \textbf{s}) \Bigg), \end{aligned}$$

where $\hat{p}_c (\textbf{y}_t | \textbf{y}_{1:(t-1)})$ is estimated using (12), and $\textbf{s} = \left( \dfrac{\lambda S_{\text {eff}, 1}}{\sum _{c=1}^C S_{\text {eff}, c}}, \dots , \dfrac{\lambda S_{\text {eff}, C}}{\sum _{c=1}^C S_{\text {eff}, c}}\right)$, with $S_{\text {eff}, c}$ denoting the effective sample size for chain *c*; see Appendix A.4 for further details. As the MCMC samples for the PNARM model (4) are vectors of parameters, the effective sample size of a chain *c*, $S_{\text {eff}, c}$, was approximated as the median effective sample size of the sampled node coefficients. The hyperparameter $\lambda$, which governs the strength of the regularisation, was set as 1.001 following Yao et al. (2022), as $\lambda >1$ means that (13) is strictly convex in $\textbf{w}$ (although ideally $\lambda$ could be further tuned via an additional nested cross-validation step).

Given weights $w_1, w_2,..., w_C$ for samples from the *C* chains of length *M* indexed by {$\varvec{\theta }^{(m, c)}\}$, a weighted Monte Carlo estimate for the mean node *i* coefficients (10) is given by:

14 $$\begin{aligned} \frac{1}{M} \sum _{c=1}^C w_c \sum _{m=1}^M \varvec{\theta }_{z_i^{(m, c)}}^{(m, c)} \end{aligned}$$

and similarly, a weighted estimate for the posterior predictive distribution (12) is

15 $$\begin{aligned}&\hat{p}({\textbf{y}}_{T+1} | {\textbf{y}}_{1:T}) \nonumber \\&\quad = \frac{1}{M} \sum _{c=1}^C w_c \prod _{i=1}^N \sum _{m=1}^M\nonumber \\&\quad \text {Poisson}\left( y_{i, T+1}; \theta _{1, z_i^{(m, c)}}^{(m, c)} v_i + \theta _{2, z_i^{(m, c)}}^{(m, c)} x_{i, T} + \theta _{3, z_i^{(m, c)}}^{(m, c)} y_{i, T} \right) . \end{aligned}$$

Further specifics regarding the number of MCMC samples and diagnostics are provided in Appendix A.6.

### Model comparisons

A key role of time series models is to forecast future values. Taking into account the differences in response magnitudes, we can evaluate the accuracy of the point forecasts through the scaled error:

#### Definition 3

(*Scaled error* (Hyndman and Koehler 2006)) Suppose that $\hat{Y}_{i, T}$ is the point forecast for $Y_{i, T}$. The scaled error of the point forecast is

16 $$\begin{aligned} q_{i, T} = \frac{Y_{i, T} - \hat{Y}_{i, T}}{\frac{1}{T-1} \sum _{t=2}^T |Y_{i, t} - Y_{i, t-1}|}. \end{aligned}$$

The mean absolute scaled error (MASE) is defined as

17 $$\begin{aligned} \frac{1}{N |\mathcal {T}|}\sum _{i=1}^N \sum _{T \in \mathcal {T}} |q_{i, T}|. \end{aligned}$$

In our evaluations, we obtain a test MASE by taking $\mathcal {T} = \{25\}$, i.e. the data from weeks 1–24 are used to determine the scale to evaluate the error at predicting the data for week 25.

In Czado et al. (2009), tools for evaluating models for count data are discussed, recommending that predictive distributions should “strive to maximise the sharpness of the predictive distributions subject to calibration". In the continuous setting, calibration can be evaluated by comparing the probability integral transforms (PIT), which is the value that the predictive cumulative distribution function (CDF) attains at the observation, to the standard uniform distribution. However, for discrete distributions the probability integral transform under the true predictive distribution is not standard uniform, due to the discontinuities in the CDF of a discrete distribution. The randomised PIT was introduced to smooth out these jumps (Czado et al. 2009):

#### Definition 4

*(Randomised probability integral transform)* Let $P^{(i)}$ be the predictive CDF for a (scalar) random variable $X_i$ and let $V_i \sim Uniform (0, 1)$ independently of $X_i$. Then a randomised PIT for the observed value $x_i$ is

18 $$\begin{aligned} U_i = P^{(i)}(x_i-1) + V_i (P^{(i)}(x_i) - P^{(i)}(x_i-1)). \end{aligned}$$

Under the null hypothesis that the predictive distribution is correct, the randomised PITs are i.i.d. standard uniform, and hence the calibration of a model can be assessed by checking independence and uniformity of the randomised PITs across all the $i \in \mathcal {I}$ observations (Brockwell 2007).

This is more complicated when our random variables are $\textbf{Y}_{t}$ vectors. If $Y_{1, t}, Y_{2, t}, \dots , Y_{N, t}$ are independent conditional on $\mathcal {F}_{t-1}$, the density estimate can be decomposed to derive expressions as in (18). However, for the Bayesian mixture models such as (4), as the predictive distribution in (11) suggests, $Y_{1, t}, Y_{2, t}, \dots , Y_{N, t}$ are not independent conditional on $\mathcal {F}_{t-1}$ because of the dependencies from the sharing of $\varvec{\theta }$ coefficients in a cluster. Nevertheless, we can still use the marginal predictive CDFs to form randomised PITs that can serve as indicative diagnostics, especially if the dependence is weak.

The ‘sharpness’ of a predictive distribution refers to its concentration: more predictive mass at the observed value is preferred. Sharpness can be evaluated through a mean *score* (Czado et al. 2009), such as

19 $$\begin{aligned} \frac{1}{N |\mathcal {T}|}\sum _{i=1}^N \sum _{t \in \mathcal {T}}\left( -\log (P^{(i, t)}(y_{i, t}) - P^{(i, t)}(y_{i, t} - 1)) \right) , \end{aligned}$$

where $y_{i,t}$ is the value observed for $Y_{i,t}$;  smaller scores indicate better predictive performance. While Czado et al. (2009) also suggest several other scoring rules, we will consider the logarithmic score for ease of computation, as it is a function of only the predictive probability mass function evaluated at the observed value. For comparison with the Gaussian-distributed GAGNAR model, we use a continuity correction, i.e. use $\log (P^{(i, t)}(y_{i, t}+0.5) - P^{(i, t)}(y_{i, t} - 0.5))$ in (19), where the predictive distribution is approximated using the MCMC samples in an analogous way to (12). Here we use the score (19) for both the training set constituted by the data of weeks 2–24 (the ‘Training score’ assesses how well weeks 1–23 predict weeks 2–24) and the test set constituted by the data of the last week, week 25 (‘Test score’). The smaller the score, the better.

**Fig. 5:**
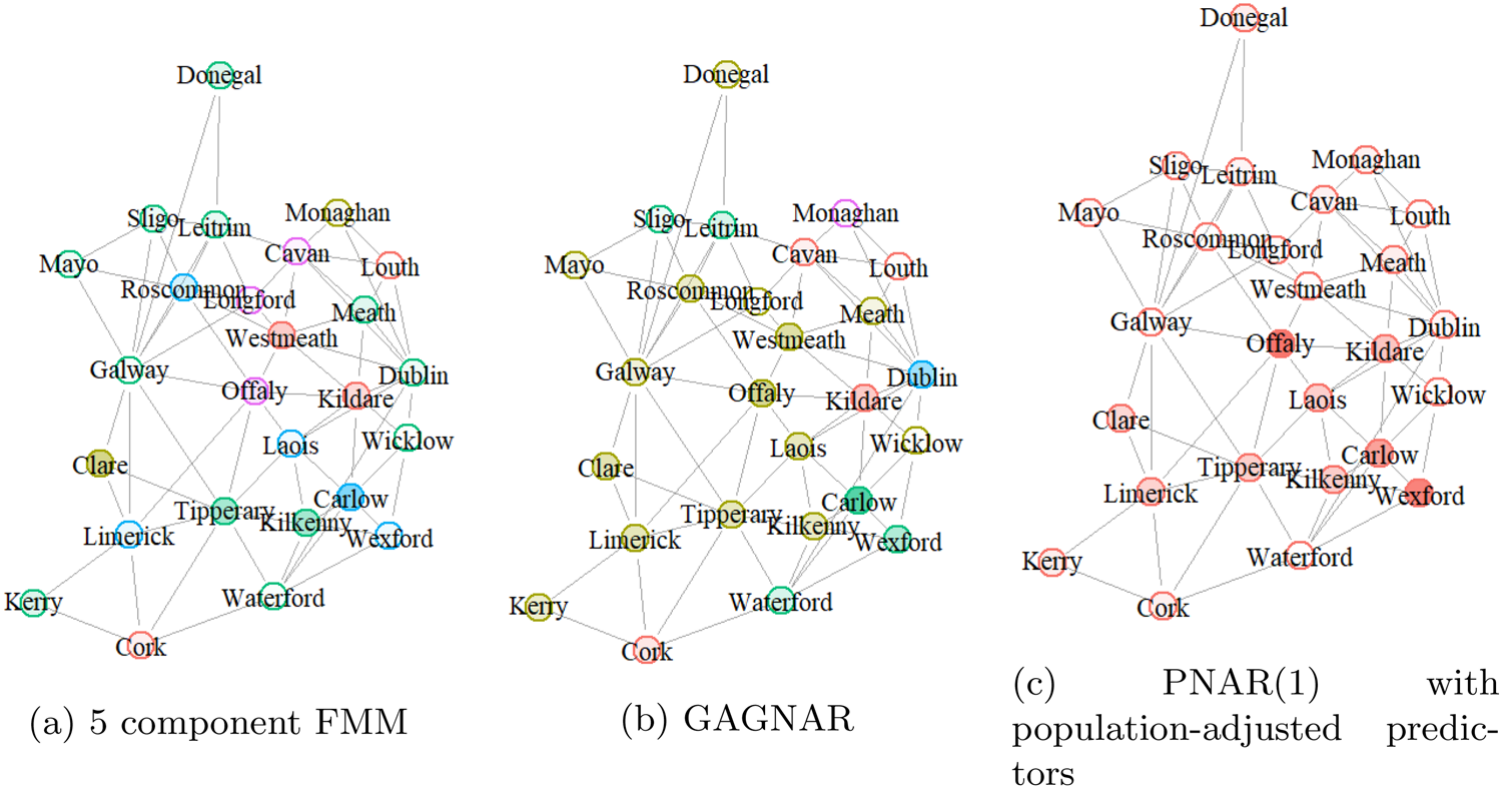
Magnitudes of scaled errors from forecasts: colours correspond to least-squares partitions from the 5-component mixture model and GAGNAR models and higher opacity reflects higher scaled error in the point forecast. Note that there is no clustering for the PNAR model

Table 5 compares the predictive performance of the PNARM models to the GAGNAR and PNAR models on the COVID-19 data, also providing a comparison to the GAGNAR model fitted to a differenced series, and the PNAR model (1) using a log-linear link, while Fig. 5 gives a visualisation of the magnitudes of the mean absolute scaled errors, across different counties and the clusters obtained under different models. There seems to be a correspondence between counties with very large or very small ratios of the values observed in week 24 versus week 25 and counties with large prediction errors. From preliminary MCMC runs, other DDP hyperparameter values of ${(}\alpha , h {)}$ seemed to produce similar posterior distributions for the coefficients (see Appendix A.2), so we do not investigate the choice of prior on the coefficients further.

**Table 5:** Performance of different models applied to the Ireland COVID-19 data,evaluated by MASE (17) (for point t = 25), training score (19) (based on t =2, . . . , 24) and test score (based on t = 25). The PNARM models used a Γ(1, 1)prior for the coefficients. The model with the best metric is in bold. More details on each model are provided in Appendix A.5.

Model specification	Mean abs. scaled error	Training score	Test score
PNARM, DDP prior with $h=1$, $\alpha =1$	0.52	**6**.**23**	**4**.**47**
PNARM, 5-component FMM	**0**.**46**	6.42	4.81
PNARM, 4-component FMM	0.50	6.53	5.09
PNARM, 1 component FMM	0.68	10.85	6.62
GAGNAR	0.65	6.99	6.99
GAGNAR with differenced series	0.74	6.68	6.59
PNAR with raw counts	0.70	7.07	6.52
PNAR with log-linear link	0.86	7.41	7.22
PNAR with population-adjusted			
predictors	0.68	7.13	6.17

Our work suggests that the PNARM(1) models outperform the GAGNAR(1) and PNAR(1) models on the Ireland COVID-19 data set, showing the utility of having network time series models tailored for count distributions that allow for heterogeneous node behaviour.

We observe that the Adjusted Rand Index comparing the least-squares partition obtained by the 5-component and GAGNAR models is 0.31; we conjecture that the differences in the partitions could arise because the GAGNAR model assumes constant innovation variance within a cluster, unlike the PNARM model.

To assess calibration of the models, we computed randomised probability integral transforms (PIT), which under the true data-generating process, should be standard uniform. However, histograms of the randomised PITs of the training data exhibited U-shapes, suggesting overdispersion relative to the models with Poisson distribution, while the histogram for the GAGNAR model had PITs concentrated around the 0.5 region (results not shown).

## Discussion and future work

In this paper, we proposed an extension to network count time series models that follow a Bayesian approach. We envisage that the posterior distributions derived for the Ireland COVID-19 data could be used in future studies of COVID-19 data.

While our analysis has shown that a Poisson error model can be more appropriate than a Gaussian error model for count network time series analysis, other response distributions may also be appropriate, such as a binomial response; for the COVID-19 application, a Pólya-Aeppli process could be investigated to take into account the COVID-19 cases that occur as a cluster outbreak (Health Protection Surveillance Centre 2020).

Furthermore, while our results show some robustness towards the choice of the underlying given network, a drawback of the PNARM model is that it depends on a known network structure, so a misspecified network structure may introduce bias when estimating coefficients. As a direction for future work, the network structure could be determined in a data-driven way by using a Bayesian modelling approach to allow for some flexibility with respect to prior specification for the network. Inspired by the approach in Corneck et al. (2024) who assume an unknown adjacency matrix, we could assign a prior distribution on the possible network structures to account for the uncertainty in the network structure. An alternative way of accounting for the uncertainty in the network structure is to assume that the observed adjacency matrix $\varvec{A}$ is a noisy realisation of a true, non-observed adjacency matrix $\varvec{A}_\text {true}$, borrowing ideas from Le and Li (2022).

Another limitation of more complex hierarchical models, such as the PNARM model, in comparison to simpler alternatives are potential MCMC mixing issues from sampling over a more complicated parameter space where there is high posterior correlation between the parameters. This is further exacerbated by having the number of parameters grow with the number of time series as in the DDP prior. Therefore, more computationally efficient methods would be needed to work with large scale networks. However, in the context of smaller-scale data sets where the primary objective is to uncover latent behaviour across time series, the flexibility that the PNARM model provides may be preferred to simpler alternatives, especially when the simpler models are unable to distinguish cross-series variability.

Currently, for predicting a new time point, the PNARM model has to be fitted again on the whole data set, which may not be very efficient. An important extension would be to develop techniques for a recursive update to the posterior distribution, allowing for more efficient handling of streaming data.

## Figures and Tables

**Fig. 6:**
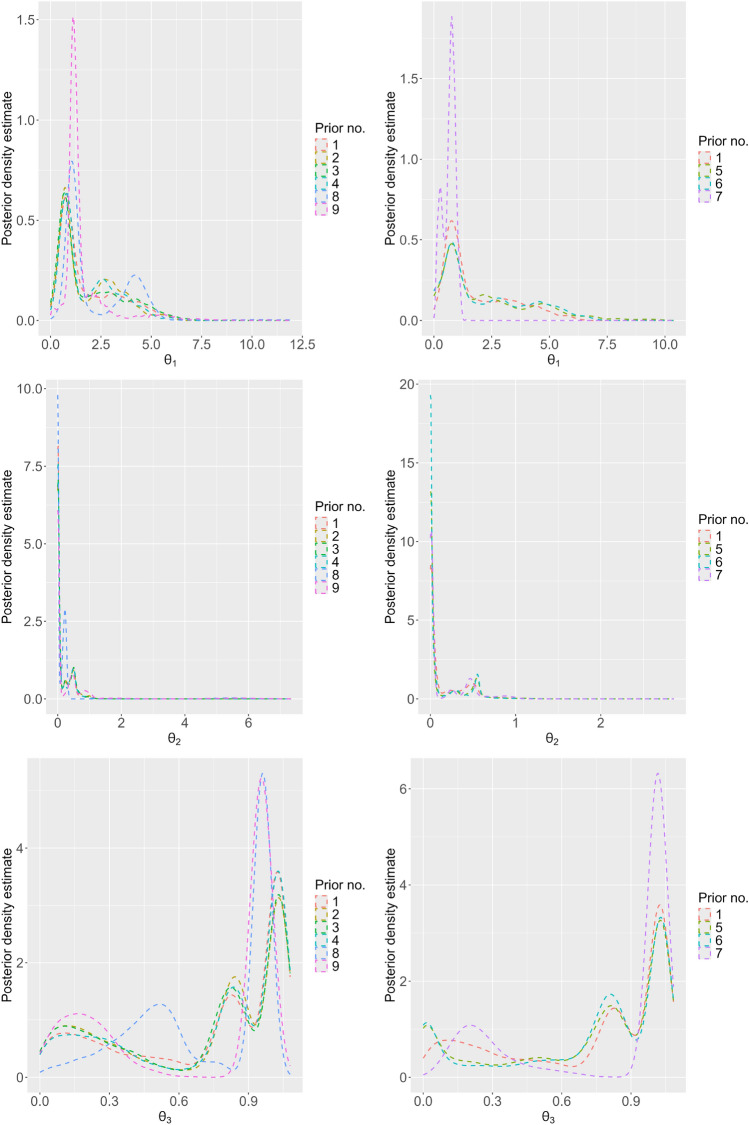
Posterior distributions of node coefficients across all nodes, under different priors numbered according to Table 6, for simulated data

**Fig. 7:**
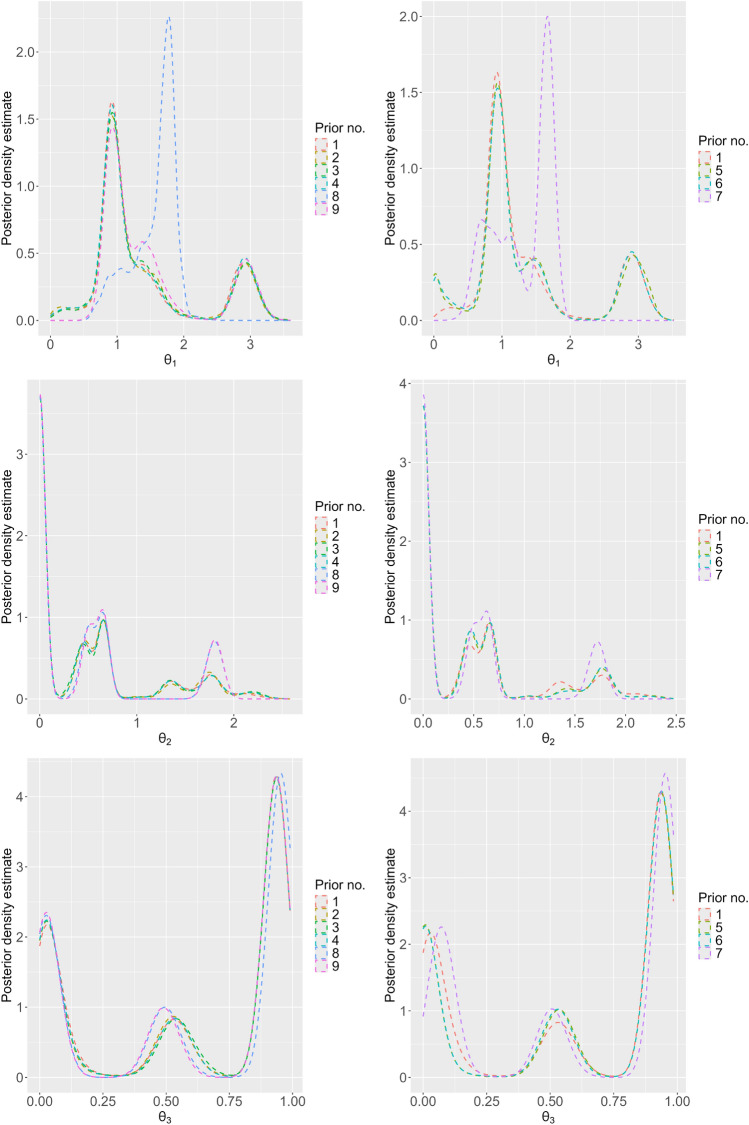
Posterior distributions across all node coefficients under different priors (from Table 6, but now prior 9 is the 5-component mixture) on the data

**Fig. 8:**
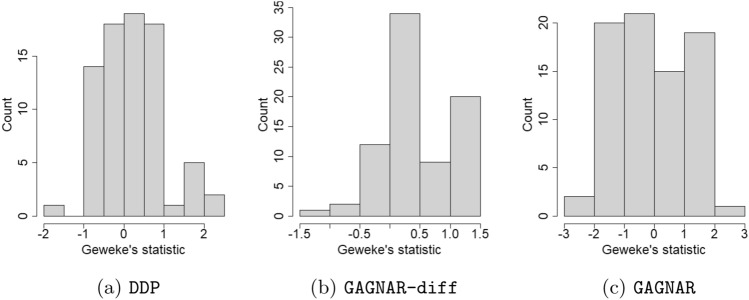
Distribution of Geweke’s test statistic for different node coefficients, from the MCMC samples used for different models

**Fig. 9:**
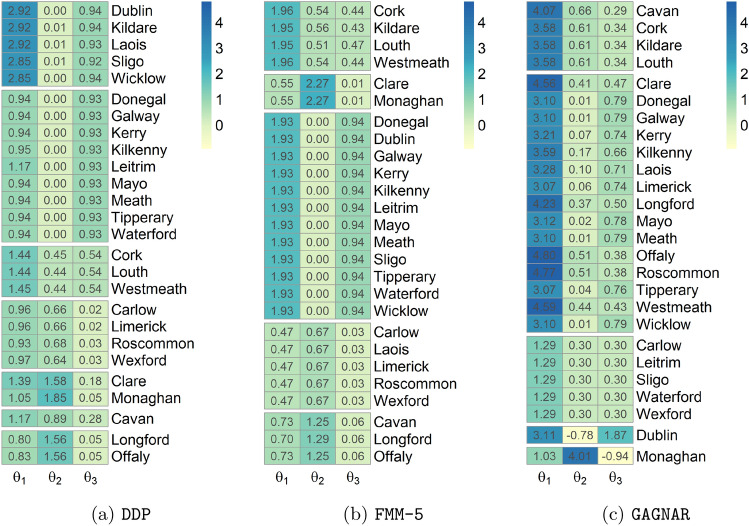
Posterior mean node coefficients (10) under different priors, grouped by a least-squares partition

## Appendix

### Proof of Proposition 2

The sufficient condition for stationarity is derived as a special case of the following result from Debaly and Truquet (2021).

#### Proposition 5

(Corollary of Theorem 2 in Debaly and Truquet (2021)) A sufficient and necessary condition for a Poisson Vector Autoregression model of the form

20 $$\begin{aligned} \mathbf {Y_t} | \mathcal {F}_{t-1} \sim \text {Poisson}(\mathbf {\mu + BY_{t-1}}) \end{aligned}$$

where $\mu \in \mathbb {R}^n$ and $\textbf{B} \in \mathbb {R}^{n \times n}$ to be stationary is for the spectral radius $\rho (\textbf{B}) < 1$.

#### Proof

Let $\textbf{D}$ be the diagonal matrix of node degrees, $\textbf{A}$ the adjacency matrix of the graph, and $\mathbf {C_k} := \text {diag}(\textbf{1}_{z_1=k}, \dots , \textbf{1}_{z_N=k})$, and define $\Vert \textbf{M}\Vert _\infty := \sup _{i \in \{1, \dots , n\}} \sum _{j=1}^n |M_{ij}|$ for any $\textbf{M} \in \mathbb {R}^{n \times n}$.

We first consider the case $X_{i, t-1} = \frac{1}{\text {deg}(i)} \sum _{j=1}^N A_{i, j} Y_{j, t-1}$. We rewrite the VAR model (20) with our community and network restrictions by setting $\mathbf {B = B_2 D^{-1} A + B_3}$, where $\mathbf {B_2} := \sum _{k=1}^K \theta _{2, k} \mathbf {C_k}$ and $\mathbf {B_3} := \sum _{k=1}^K \theta _{3, k} \mathbf {C_k}$ are both diagonal matrices. Then

$$\begin{aligned} \begin{aligned} \rho (\textbf{B})&\le \Vert \mathbf {B_2 D^{-1} A + B_3}\Vert _\infty \\ &= \max _{i=1,...,N} \left| \theta _{2, z_i} \frac{1}{\text {deg}(i)}\sum _{j=1}^N A_{ij} + \theta _{3, z_i} \right| \\ &= \max _{k=1,...,K} \theta _{2, k} + \theta _{3, k}, \end{aligned} \end{aligned}$$

as we assume that the coefficients are positive.

For the population-adjusted model, we may bound the operator norm by

$$\begin{aligned} \begin{aligned} \Vert \textbf{B}\Vert _2&\le \Vert \mathbf {B_2}\Vert _2 \, \Vert \mathbf {D^{-1} P A P^{-1}}\Vert _2 + \Vert \mathbf {B_3}\Vert _2 \\ &= \max _{k = 1,...,K} \theta _{2, k} \, \sqrt{\rho ((\mathbf {D^{-1} P A P^{-1}})^\top \mathbf {D^{-1} P A P^{-1}})} + \max _{k = 1,...,K} \theta _{3, k} \\ &\le \max _{k = 1,...,K} \theta _{2, k} \, \sqrt{\rho (\mathbf {P^{-1} A D^{-1} P})} \, \sqrt{\rho (\mathbf {P D^{-1} A P^{-1}})} + \max _{k = 1,...,K} \theta _{3, k} \\ &\le \max _{k = 1,...,K} \theta _{2, k} \, \sqrt{\rho (\mathbf {A D^{-1}})} \, \sqrt{\rho (\mathbf {D^{-1} A})} + \max _{k = 1,...,K} \theta _{3, k} \\ &\le \max _{k=1,..., K} \theta _{2, k} + \max _{k=1,..., K} \theta _{3, k}, \end{aligned} \end{aligned}$$

since diagonal matrices commute, similar matrices have the same eigenvalues, and the spectral radius of a stochastic matrix is at most 1. This finishes the proof. $\square$

### Coefficient prior sensitivity

To investigate the sensitivity of the posterior distribution to the priors over the partition and coefficients, we consider the different priors specified in Table 6. Since the autoregressive coefficients are restricted to be positive for a linear model to ensure that the expectation is positive, we model the coefficients as being drawn from $\Gamma (a, b)$ priors. The prior mean is *a*/*b* and the prior variance is $a/b^2$, so a larger $a/b^2$ value corresponds to being less certain about the magnitudes of the coefficients. All coefficients are assumed to be drawn from the same prior, so it is uninformative with respect to the magnitudes of coefficients from different clusters and coefficient types (baseline, network effect, nodal effect).

**Table 6:** Priors considered, with $h_{ij} = \exp (-h \times d_{ij})$ in the distance-dependent partition prior

No	Partition prior	Parameter prior	Prior information relative to (1)
(1)	DDP, $\alpha =1, h=1$	$\Gamma (1, 1)$	
(2)	CRP, $\alpha =1$	$\Gamma (1, 1)$	Uniform co-clustering
(3)	DDP, $\alpha =1, h=1/4$	$\Gamma (1, 1)$	Increase co-clustering with neighbours
(4)	DDP, $\alpha =1/4, h=1$	$\Gamma (1, 1)$	Fewer clusters expected
(5)	DDP, $\alpha =1, h=1$	$\Gamma (1/4, 1/4)$	$4 \times$ coefficient variance
(6)	DDP, $\alpha =1, h=1$	$\Gamma (1/4, 1/2)$	Halve coefficient mean
(7)	DDP, $\alpha =1, h=1$	$\Gamma (4, 16)$	Strongly informative for coefficients
(8)	Dirichlet(1, 1, 1, 1)	$\Gamma (1, 1)$	Non-informative partition into 4 clusters
(9)	Dirichlet(1, 1, 1)	$\Gamma (1, 1)$	Non-informative partition into 3 clusters

Figure 6 shows plots of posterior coefficient distributions (across all nodes) for a length 25 time series simulated on the economic hubs network using the Example 3 coefficients from Table 1, as this is the most relevant configuration for our data application. In general, it seems that for a time series of this length, size and regime, the results are not sensitive to small changes in the $(\alpha , \beta )$ hyperparameters of the DDP prior, as well as to small changes in the shape, rate parameters of the Gamma prior for the coefficients, under the DDP prior for the partition. Prior 7 is included for a comparison with a more strongly informative prior for the coefficients, which a practitioner may choose if they have prior beliefs about the node dynamics – we see that it still allows for some updating of the posterior based on the data, but results in a more concentrated posterior distribution.

### Network sensitivity

To assess the impact of network choice on modelling the Ireland COVID-19 data, we also fit the PNARM model with a DDP prior to a complete network (where all edges in the graph are present) and the ‘railway-based network’ from Armbruster and Reinert (2024). In the railway-based network, there is an edge between two counties if and only if there is a direct train link between the county towns and they are closest to each other on this train connection. The railway-based network is sparser than the economic hubs network and has a larger average shortest path length. The results on the MASE, the training score, and the test score of the PNARM model on these networks are collected in Table 7. While the PNARM model on the economic hubs network has smallest MASE and test scores, the PNARM model on the railway-based network shows similar performance. In contrast, the PNARM model on the complete network does not explain the data as well. This finding illustrates some robustness regarding network choice while underlining that the sparsification achieved via a non-complete network is advantageous.

**Table 7:** Performance under different choices of network, evaluated by MASE (17) (for point $t=25$), training score (19) (based on $t=2, \dots , 24)$ and test score (based on $t=25$)

Model specification	MASE	Training score	Test score
Economic hubs network	0.52	6.23	4.47
Railway-based network	0.53	6.19	4.59
Complete network	0.74	6.30	6.10

### Some notions from Bayesian analysis and MCMC

Bayesian statistics is a framework for inference based on Bayes’ theorem. A typical Bayesian workflow begins by eliciting a distribution, known as a *prior* distribution $\pi (\theta )$, to capture knowledge about a parameter $\theta$ of a model with likelihood $f( \cdot ; \theta )$. After observing some data *X*, by Bayes’ rule, the *posterior* distribution of the parameter is updated to be

21 $$\begin{aligned} \pi (\theta \mid X) = \frac{\pi (\theta ) f(X; \theta )}{p(X)} \propto \pi (\theta )f(X; \theta ). \end{aligned}$$

Based on the posterior distribution and the likelihood, a *posterior predictive* distribution can be constructed for future observations (Gelman et al. 2020).

It is often difficult to evaluate (21) directly, leading to the use of Markov Chain Monte Carlo (MCMC) methods to target samples from (21) to perform inference. We give a brief introduction to some concepts in practical MCMC that have been referred to in this paper below:

- *Burn-in*: since the convergence of an MCMC algorithm to the stationary distribution is asymptotic, some samples tend to be discarded from the beginning of the chain where it has not stabilised to the stationary distribution.
- *Effective sample size*: MCMC samples are not independent as they are generated from a first-order Markov Chain. The effective sample size approximates how many independent samples contain the same amount of information as the MCMC samples, by adjusting for (an approximation of) the autocorrelation.
- *Geweke’s test statistic:* is used as an informal check for whether the MCMC chain has converged to its stationary distribution. Geweke’s statistic is based on the two-sample *Z*-test: taking samples from the beginning and end of the chain, the difference of the means divided by the square root of estimates of the sample variances (adjusted for autocorrelation). Under the null hypothesis that both parts of the chain have the same distribution, the test statistic is asymptotically standard normal (Geweke 1992).

### Models considered

Since the motivation for the PNARM model is to model heterogeneous count time series on the nodes of a network, we compare our model to other network autoregression models: the GAGNAR model given in (2) (which is also a mixture model but not for count responses) and PNAR models (which are for count responses but only use one set of coefficients and thus cannot model heterogeneous node behaviour). The models are as follows.

- DDP: A PNARM(1) model, where the partition prior $\pi _Z$ is the Dahl (2008) prior with $\alpha =1$ and $h_{ij} \propto \exp (-d_{ij})$ where $d_{ij}$ is the shortest network path length between two nodes, and and $\theta _{l, k} \sim \Gamma (1, 1)$ for all *l*, *k*. The choice of $\alpha =1$ and unit scaling in the exponent follows from an informal prior sensitivity analysis in Fig. 7, suggesting that the posteriors would otherwise not be very different.
- FMM-*K* : A PNARM(1) model, where $\pi _Z$ is a *K*-component mixture model, with noninformative Dirichlet(1,..,1) prior for the mixture proportions, and $\theta _{l, k} \sim \Gamma (1, 1)$ for $l=1, 2, 3$ and $k = 1, \dots K$. The PNARM FMM-1 model is equivalent to a PNAR model with a $\Gamma (1, 1)$ prior on the coefficients.
- GAGNAR: This fits the GAGNAR model (2), using the same non-informative hyperparameters that have been used throughout the Ren et al. (2024) paper of $a_0 = b_0 = 0.01$, $\mu _0 = \textbf{0}$, but with a lower prior variance for the coefficients of $\mathbf {\Sigma _0 = I_3}$ (as opposed to their choice of 100$\mathbf {I_3}$). Informally conducted trial runs of the MCMC algorithm under different priors did not suggest there would be large differences in the posteriors.
- GAGNAR-diff: Following (Armbruster and Reinert 2024), we fit the GAGNAR model, with hyperparameters as above, to the multivariate time series $\mathbf {U_t = P^{-1}(Y_t - Y_{t-1})}$, so that it is closer to the assumptions of stationarity and isotropic Gaussian errors. A point forecast can be recovered as ${\hat{\textbf{Y}}_\textbf{T} = \textbf{Y}_{\textbf{T}-1} + \textbf{P} \hat{\textbf{U}}_\textbf{T}}$. Posterior samples for the two GAGNAR models were obtained using the code provided in the supplementary material to Ren et al. (2024).
- PNAR: A linear PNAR(1) model (1) is fitted to the number of weekly cases. The model coefficients are estimated using the lin_estimnarpq() function in the PNAR package (Armillotta et al. 2024) in R.
- PNAR-ll: A log-linear PNAR(1) model, as in Armillotta and Fokianos (2024) is fitted to the number of weekly cases. The model coefficients are estimated using the log_lin_estimnarpq() function in the R package PNAR.
- PNAR-adj: A linear PNAR(1) model but with population-adjusted predictors, to try to distinguish between the impacts of node-varying coefficients and population-adjusted predictors on model performance.

### MCMC diagnostics

For approximations using MCMC samples, such as (12), to be valid, we require the MCMC to be converging to the posterior distribution. One way of assessing convergence is to run multiple chains that are initialised at different starting points, and to see if they converge to the same distribution (Gelman et al. 2013).

For the DDP prior, the MCMC algorithm returns similar posterior densities across 10 different initialisations of the sampler, which is not the case for the samplers when considering a finite mixture prior. Therefore, any conclusions drawn based on the MCMC samples for the finite mixture model could be highly dependent on where the sampler was initialised. The lack of mixing could not be resolved with common fixes such as changing the random-walk step size or running the chains for a (reasonable) longer number of iterations, so for the finite mixture models, we obtain samples using the chain-stacking approach described in Sect. 5.4. More specifically,  five chains were run for 25 000 iterations each using the data from time points $t=2,...,20$, discarding the first 5000 iterations of each chain as burn-in. Then, the samples were pooled by computing optimal weights as described in (13), using $t=21,...,24$ as the validation data.

For the remaining models fitted using MCMC, the samples were obtained from a single chain. The MCMC was run for 100,000 iterations for the DDP and GAGNAR-diff models, and for 150,000 iterations for the GAGNAR model.

Geweke (1992) proposed a test for stationarity by testing whether the first and last parts of the chain have the same mean, with the first and last parts taken here to be the first 10% and last 50% of the (not discarded) samples from the chain respectively. This test was used to determine how many samples to discard from the chains as burn-in: discarding the first 5000, 10 000, and 65 000 samples obtained from the samplers of the DPP, GAGNAR, and GAGNAR-diff models, respectively, results in the Geweke statistics for the node coefficients shown in Fig. 8, where we see that most of the values lie between −2 and 2. These values are in line with typical values from a standard normal distribution.

### Additional results for the COVID-19 data application

Figure 9 shows the posterior mean node coefficients (10) for different models, grouped by a least-squares partition determined by (9). There are some similarities between the mean node coefficients obtained under the DDP and FMM-5 models as might be expected; we see that the FMM-5 model almost merges some of the clusters obtained from the DDP model and takes the average from the coefficients. For example, in the DDP model, the {Dublin,..., Wicklow} cluster and the {Donegal,..., Waterford} cluster have mean $\theta _1$ coefficients of around 2.9 and 0.95 respectively, and both clusters have similar mean $\theta _2, \theta _3$ coefficients of 0 and 0.93. These two clusters seem to be merged into one cluster in the FMM-5 model with mean cluster coefficients of $(\theta _1, \theta _2, \theta _3)$ = (1.93, 0.00, 0.94). For comparison, the coefficients obtained under the PNAR-adj model, where all counties are in the same cluster, are (1.79, 0.00, 0.95).

There are more differences between the coefficients of the PNARM models and the GAGNAR model. We conjecture that perhaps in the GAGNAR model the coefficients are pulled towards an average of nodes that have similar innovation variances.

Some of the coefficients for Dublin and Monaghan, which form singleton clusters, are negative. The negative coefficients may be due to multicollinearity – the average Pearson correlation coefficient for the predictors $({\textbf{X}}_{i}, {\textbf{Y}}_{i})$ across all counties *i* is 0.84, where ${\textbf{X}}_{i}$ is a vector of scaled weighted average of case counts in counties that share an edge with *i*, and ${\textbf{Y}}_{i}$ is the vector of case counts in *i*.
